# Ischemic Stroke in Confederation with Trivial Head Trauma

**DOI:** 10.1155/2016/2572958

**Published:** 2016-05-29

**Authors:** Archana Balachandran, Siddarameshwar Kalyanshettar, Shankargouda Patil, Vijaykumar Shegji

**Affiliations:** Department of Pediatrics, Shri. B.M. Patil Medical College Hospital and Research Centre, BLDE University, Vijayapur, Karnataka 586103, India

## Abstract

Minor head injuries in children are common, resulting in brain concussion, and these injuries mostly end up without complications. Usually head trauma results in hemorrhagic stroke. Here we present a case of ischemic stroke following a trivial head trauma. A 10-month-old girl presented with posttraumatic right sided hemiparesis with right sided facial palsy. MRI brain revealed an area of acute infarct in the left capsuloganglionic region. The child was initially managed conservatively, as the hematological parameters were normal, and was started on anticoagulant therapy. An improvement in the clinical condition was achieved in 12 hrs of treatment with gain in power and resolution of weakness in 10 days. The specific cause for hemiparesis in the child is not elicited; possibility of genetic and environmental factors can be attributable.

## 1. Introduction

Trivial head injuries are the most common accidents in childhood. They most often present with normal neurological findings. The severity of the head trauma correlates with the clinical symptoms. Cranial trauma in young children can cause ischemic infarct [1]. Incidence of cerebrovascular disease in children in India is 13–33/100,000 per year [2]. Head trauma in children mostly results in hemorrhagic stroke and ischemic stroke, of which ischemic cause is of lesser frequency. Hemorrhagic strokes are usually common in preceding vascular or coagulation anomaly [3]. Ischemic stroke constitutes slightly more than 50% of childhood and 80–85% of adult stroke cases [4]. Head trauma appears to act as a trigger for arterial strokes and dehydration for venous strokes [5]. The occurrence of ischemic lesion in the basal ganglia is a rarely described event following a minor head trauma with an incidence of less than 2% of all ischemic strokes in childhood [3]. The occurrence of ischemic lesions confined to the subcortical region is uncommon [6], and despite several case reports and descriptions a complete analysis of this illness in childhood is not available. Usually, mild pediatric stroke cases will have a good prognosis and complete recovery by proper management.

Here we report a case with neurological deficit following a minor head injury probably due to postbleed thrombosis resulting in ischemia of small vessel territories.

## 2. Case Report

**Figure 1:**
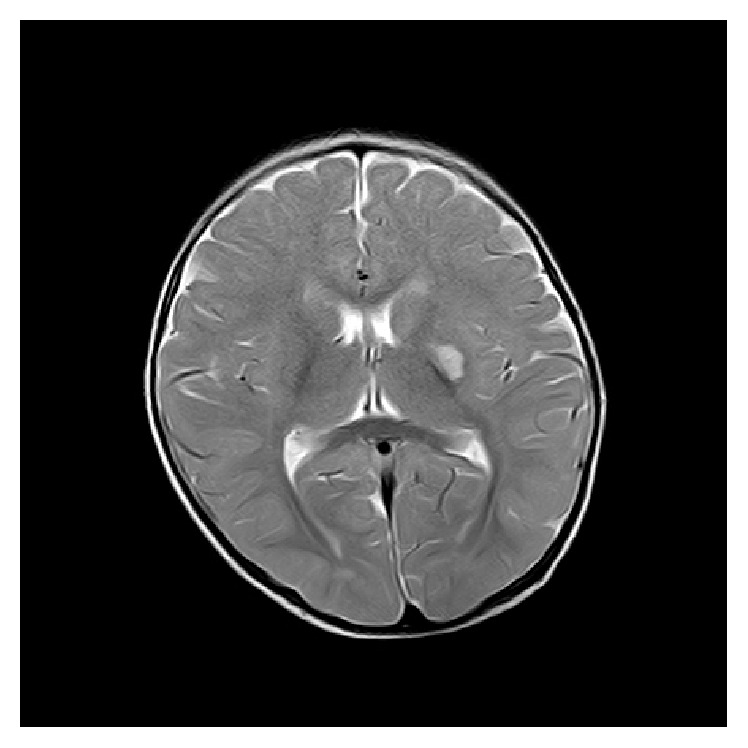
T2 weighted imaging of brain showing acute infarct of left capsuloganglionic region.

**Figure 2:**
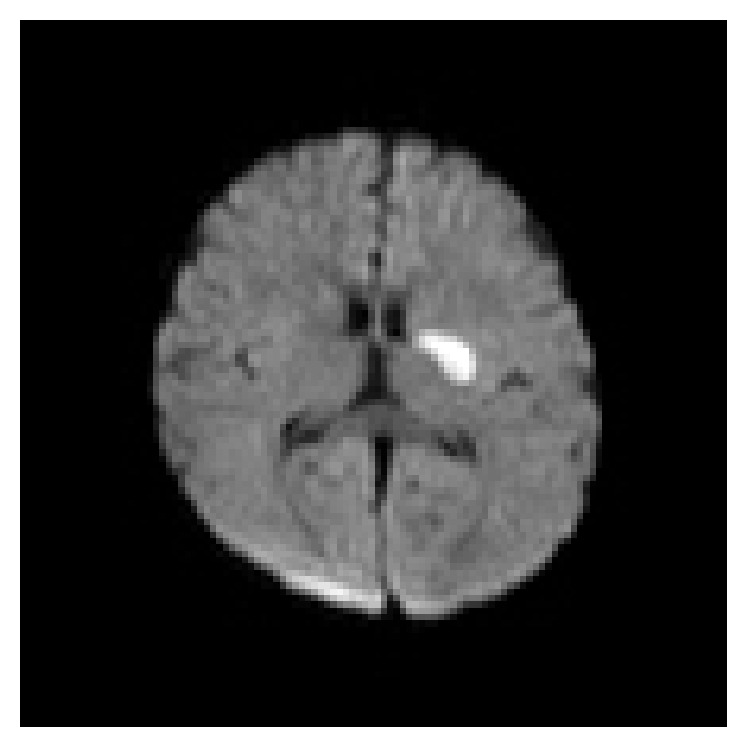
Restricted diffusion on DWI noted in left capsuloganglionic region suggestive of acute infarct.

A 10-month-old girl born of nonconsanguineous marriage with normal developmental history, with no prior medical problems, was brought to our emergency room with history of fall on the head when the child was trying to walk on her own. She cried for a brief period and there was no loss of consciousness. There was no vomiting or convulsions following the head injury. Next day morning, about 12 hours after the injury, child developed weakness involving right side of the face, right arm, and the right leg. Child was unable to sit or stand. On examination child was conscious and active showing decreased movements in the right half of the body with muscle power 2/5 in both right upper and lower extremity along with right sided UMN type of facial palsy, with a positive Babinski sign on the same side. There were no signs of raised intracranial pressure or meningeal irritation. Other neurological examinations were normal. Child did not have any signs suggestive of nonaccidental injury and did not have any external signs of trauma to the head or elsewhere on the body. Child had no clinical history suggestive of varicella, measles, or ear discharge in the past. There was no history of feeding difficulty which suggests that there was no lower cranial nerve involvement. Perinatal history was uneventful. There were no significant family history suggestive of thromboembolic disorders and no history of recent vaccination. Investigations like routine blood count, peripheral smear, serum electrolytes, bleeding, and coagulation profile were done and found to be within normal limits. MRI brain suggested acute infarct involving the left capsuloganglionic region (Figures 1 and 2). Child was managed conservatively with osmotic diuretics to reduce perilesional oedema and also was started on anticoagulant therapy subcutaneously. Low molecular weight heparin was given for a total of 5 days and was stopped without any continuation of oral antiplatelet therapy. Parents could not afford other relevant investigations including MRA, Doppler evaluation of the vessels, lipoprotein A and homocysteine estimation, protein C and S, antithrombin III, and antiphospholipid antibody. The child started moving her right leg with gain in power after 12 hrs of anticoagulant therapy. The facial paralysis and the hemiparesis improved following 5 days of treatment and the child was discharged with advice of physiotherapy. Neurological evaluation on follow-up after 10 days of treatment showed good clinical resolution, with power 5/5 in both right upper and lower limbs with normal reflexes and no residual facial weakness.

## 3. Discussion

Minor head injuries are very common during childhood and are rarely complicated. Acute ischemic stroke most often presents as a focal neurologic deficit. Hemiplegia is the most common focal manifestation, occurring in up to 94% of stroke cases [7, 8]. Hemorrhagic strokes most commonly present as headaches or altered level of consciousness and are more likely to cause vomiting than in ischemic stroke [7–9]. In children, ischemic stroke following trivial head trauma is exceedingly rare and should be considered after systematic exclusion of other causes. The plausibility of an insult preexisting and resulting head injury may be difficult to discriminate. In our case, the child had a history of neurological signs after a fall witnessed by her parents. It is unlikely to be nonaccidental injury since there is no other supporting clinical or radiological evidence. The child presented with neurological deficits after 12 hours of a minor head injury. Kieslich et al. observed a latency of 15 min–16 days for the evolution of basal ganglia lesion following minor head injury [10].

The presentation of hemiplegia with UMN type facial palsy with no cortical signs suggests the probable location of the lesion above the level of pons in the subcortical region.

The pathophysiology of this case can be explained by the peculiarity of the blood vessels arrangement in the brain of a child compared to that of an adult. The basal ganglia and the internal capsule are supplied by the lenticulostriate arteries which are functional end arteries. They originate from the middle cerebral artery at acute angle (the angle is more acute in children) after which it follows a recurrent course and then pierces the anterior perforated substance. Therefore any motion of the brain due to head injury may disrupt these perforating branches leading to vessel damage and thereby causes a decrease in the blood flow or intimal trauma resulting in subsequent thrombosis or spasm [11]. Brain vasculature of children is more susceptible to shearing and conductive and other biomechanical forces than that of adults.

A hypothesis suggests that an injury to the middle cerebral artery leads to its spasm and associated reduction in PCO2 due to crying, resulting in narrowing of the blood vessel, thereby decreasing the blood supply and increasing the chances of thrombosis [3]. The relative delay in the occurrence of symptoms in this case may be explained on the basis of intimal injury followed by thrombus formation. Another possibility being, with an impact from a head injury the brain parenchyma moves in opposite direction, resulting in shearing and other effects on the vessels. As the angle at the origin of blood vessels from the MCA varies with age [12] children are more susceptible to ischemic stroke. This can also be the cause in this case. Similar concerns are assumed by Kieslich et al. [10].

**Figure 3:**
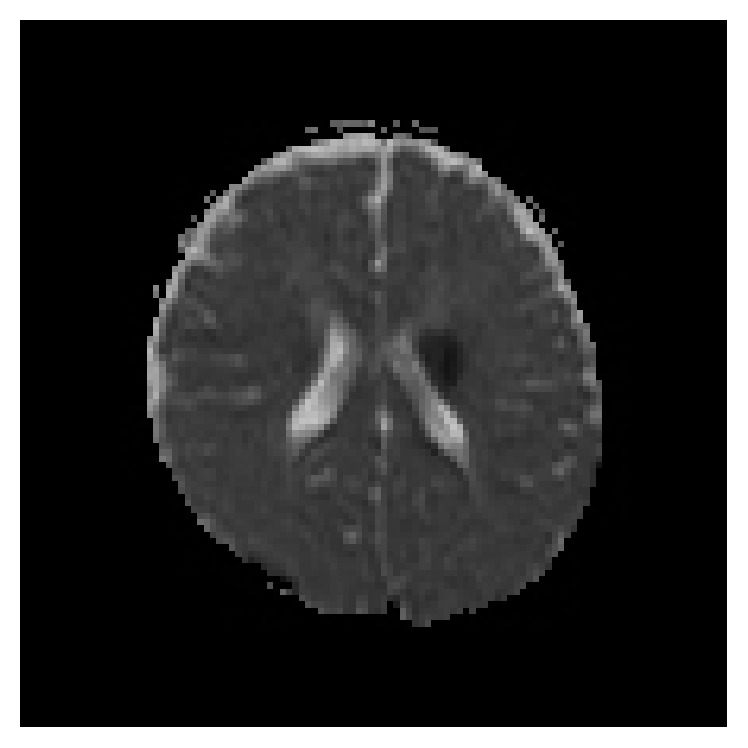
MRI brain (ADC) low signals noted in the left capsule-ganglionic region suggestive of acute infarct.

In this child, MRI brain showed acute infarct in the left capsuloganglionic region (Figure 3). This explains the clinical features that are seen.

Other acquired metabolic causes as well as genetic causes leading to thrombosis should have been ruled out. Acquired prothrombotic disorders secondary to deficiencies in protein C and S may occur in children with renal and liver disease, including nephrotic syndrome with loss of coagulation factors [4, 13]. Protein C deficiency has also been reported in children taking valproate [14]. Hemorrhagic strokes can arise from both factor VII and factor VIII deficiency [15, 16]. Iron deficiency anemia has been reported in children with both ischemic stroke and venous thrombosis with no other apparent etiology [5, 17, 18].

Among viral infections varicella is known to cause secondary vasculopathy, thus increasing the susceptibility to develop arterial thrombosis or spasm after minor head injury [19]. Varicella zoster virus reactivation is one of the potential causes of cerebral vasculopathy; for this reason, it must be considered in the differential diagnosis of pediatric transient ischemic attack and/or stroke, even when the rash is temporally remote from the acute event. However, there was no history suggestive of varicella in our case.

A genetic predisposition may also be attributed for the severity in manifestation of minor head injuries, resulting in the unfavourable outcome such as malignant syndrome which is characterized by delayed cerebral oedema and coma. A genetic mutation in the CANA1A calcium channel subunit genes was believed to be responsible for it [20].

Stroke in childhood is multifactorial. It is very important to exclude other causes of ischemic stroke in children. The common conditions predisposing to ischemic stroke include embolism due to congenital or acquired heart disease, thrombophilia and acute traumatic arterial dissections, dehydration, meningitis, acquired immunodeficiency syndrome, hemolytic uremic syndrome, homocystinuria, and syndromes like Down's syndrome and Williams syndrome [21, 22]. Other causes include sickle cell disease, which is a very common cause of pediatric stroke, occurring in 285 cases per 100,000 affected children [7]. Strokes may occur as early as 18 months of age, but most children present after five years of age [23]. Venous sinus thrombosis can present in all ages with fever and lethargy; an older child would likely present with more slowly progressing signs, such as vomiting, headache, or any other signs of increased intracranial pressure [24].

Here, the case showed no abnormality with the routine blood investigations. There are no features of anemia, high counts, and abnormal cells, thereby excluding the causes discussed above. There are no features and investigations suggestive of other organ involvement. This helped in narrowing the possibility of the cause, to be traumatic etiology.

Usually the prognosis of pediatric stroke is good. Our child showed good progress with anticoagulant therapy and had complete improvement in 10 days.

Many authors mentioned such cases in the literature like Rana et al. who presented 7 cases of ischemic stroke in minor head injury in children [25].

The clinical manifestations and the radiological features following a minor head injury, with exclusion of other causes with relevant available investigations, suggest a confederation among ischemic stroke and trivial head injuries by the aforementioned assumed mechanisms.

An elaborated history, with multidisciplinary approach in evaluation of ischemic strokes in pediatric patients with head injury, makes way for the future to prove the expected association.
